# Automated meniscus segmentation and tear detection of knee MRI with a 3D mask-RCNN

**DOI:** 10.1186/s40001-022-00883-w

**Published:** 2022-11-14

**Authors:** Yuan-Zhe Li, Yi Wang, Kai-Bin Fang, Hui-Zhong Zheng, Qing-Quan Lai, Yong-Fa Xia, Jia-Yang Chen, Zhang-sheng Dai

**Affiliations:** 1 Department of CT/MRI, The Second Affiliated Hospital of Fujian Medical University, Quanzhou, 362000 China; 2 Department of Orthopaedic Surgery, The Second Affiliated Hospital of Fujian Medical University, Quanzhou, 362000 China; 3 Institute of Biomedical Engineering and Instrumentation, Hangzhou Dianzi University, Hangzhou, China; 4 Orthopedics, Anji TCM Hospital Affiliated to Zhejiang Provincial Hospital of Traditional Chinese Medicine (Anji Traditional Chinese Medical Hospital), Anji, 313300 China; 5 Radiology Department, Anxi Hospital of Traditional Chinese Medicine, Quanzhou, 362400 China

**Keywords:** Deep learning, Meniscus tear, Magnetic resonance imaging, Segmentation, Detection

## Abstract

**Background:**

The diagnostic results of magnetic resonance imaging (MRI) are essential references for arthroscopy as an invasive procedure. A deviation between medical imaging diagnosis and arthroscopy results may cause irreversible damage to patients and lead to excessive medical treatment. To improve the accurate diagnosis of meniscus injury, it is urgent to develop auxiliary diagnosis algorithms to improve the accuracy of radiological diagnosis.

**Purpose:**

This study aims to present a fully automatic 3D deep convolutional neural network (DCNN) for meniscus segmentation and detects arthroscopically proven meniscus tears.

**Materials and methods:**

Our institution retrospectively included 533 patients with 546 knees who underwent knee magnetic resonance imaging (MRI) and knee arthroscopy. Sagittal proton density-weighted (PDW) images in MRI of 382 knees were regarded as a training set to train our 3D-Mask RCNN. The remaining data from 164 knees were used to validate the trained network as a test set. The masks were hand-drawn by an experienced radiologist, and the reference standard is arthroscopic surgical reports. The performance statistics included Dice accuracy, sensitivity, specificity, FROC, receiver operating characteristic (ROC) curve analysis, and bootstrap test statistics. The segmentation performance was compared with a 3D-Unet, and the detection performance was compared with radiological evaluation by two experienced musculoskeletal radiologists without knowledge of the arthroscopic surgical diagnosis.

**Results:**

Our model produced strong Dice coefficients for sagittal PDW of 0.924, 0.95 sensitivity with 0.823 FPs/knee. 3D-Unet produced a Dice coefficient for sagittal PDW of 0.891, 0.95 sensitivity with 1.355 FPs/knee. The difference in the areas under 3D-Mask-RCNN FROC and 3D-Unet FROC was statistically significant (*p* = 0.0011) by bootstrap test. Our model detection performance achieved an area under the curve (AUC) value, accuracy, and sensitivity of 0.907, 0.924, 0.941, and 0.785, respectively. Based on the radiological evaluations, the AUC value, accuracy, sensitivity, and specificity were 0.834, 0.835, 0.889, and 0.754, respectively. The difference in the areas between 3D-Mask-RCNN ROC and radiological evaluation ROC was statistically significant (*p* = 0.0009) by bootstrap test. 3D Mask RCNN significantly outperformed the 3D-Unet and radiological evaluation demonstrated by these results.

**Conclusions:**

3D-Mask RCNN has demonstrated efficacy and precision for meniscus segmentation and tear detection in knee MRI, which can assist radiologists in improving the accuracy and efficiency of diagnosis. It can also provide effective diagnostic indicators for orthopedic surgeons before arthroscopic surgery and further promote precise treatment.

## Introduction

As the knee joint directly affects mobility, early diagnosis and treatment of knee joint discomfort are critical. Osteoarthritis and meniscus tear are two important diseases in the knee. Osteoarthritis develops when the cartilage in the knee joint wears away, and its severity is increased by abnormalities found in the meniscus [1]. In most cases, knee pain is caused by either trauma or degeneration and meniscal tears are common findings. [2]. If the meniscus tears are untreated on time, osteoarthritis discomfort develops over time, further necessitating surgical treatment [3, 4]. Magnetic resonance imaging (MRI) plays a central role in diagnosing meniscus lesions, preoperative planning, and postoperative rehabilitation of patients [5]. In comparison with arthroscopy, MRI showed a sensitivity and specificity of 93% and 88%, respectively, for tears of the medial meniscal, and 79% and 96%, respectively, for the tears of the lateral meniscus, with an accuracy of 85–90%. However, it is still unable to replace arthroscopy as the gold standard for meniscus tears, because under arthroscopy, orthopedists can directly observe the degree of meniscus lesions and make a more intuitive diagnosis [6, 7]. The diagnostic results of MRI are important references for arthroscopy as an invasive procedure. A deviation between medical imaging diagnosis and arthroscopy results may cause irreversible damage to patients and lead to excessive medical treatment. Consequently, an accurate imaging diagnosis of a meniscus tear is critical [8]. Presently, with the explosive growth of medical imaging data, with much interference and useless information, radiologists face challenges that restrict their ability to accurately diagnose. A tool that can effectively assist radiologists in improving diagnostic accuracy is urgently required. Computer-assisted diagnostic of medical imaging systems has been proposed to detect abnormalities in the knee joint for early diagnosis and treatment purposes. Numerous radiomics algorithms and deep learning algorithms are employed for the intelligent diagnosis of meniscus lesions in medical imaging due to the advancements in artificial intelligence (AI) driven by the rise in computing power and improvement in big data management [9–14].

To our knowledge, our article presents the first example of a 3D Mask-RCNN deep convolutional neural network for automatic meniscal segmentation and detection of meniscal tears (with arthroscopy as a standard of reference). Consequently, the purpose of this study aimed to validate a fully automatic 3D-based DCNN for knee MRI to detect and segment meniscus tears, proven surgically by arthroscopy. Putting our results into the perspective of meniscal injury research and clinical application, this study sought to (a) demonstrate the performance of our knee MRI automated meniscus segmentation method compared to manual segmentation and (b) use arthroscopy as a standard, comparing the diagnostic performance of our knee MRI meniscal tear detection with radiologists. Finally, after clinical verification, the automatic 3D-DCNN segmentation detection of meniscus tear under arthroscopy can effectively improve the accuracy of meniscus tear detection, reduce unnecessary arthroscopic examinations for patients, and promote the precise treatment of meniscus tears by reducing excessive surgical treatment.

## Materials and methods

Our local ethics committee approved this retrospective study. Written informed consent for retrospective data analysis was obtained from all included subjects.

### Study design and data set

**Fig. 1:**
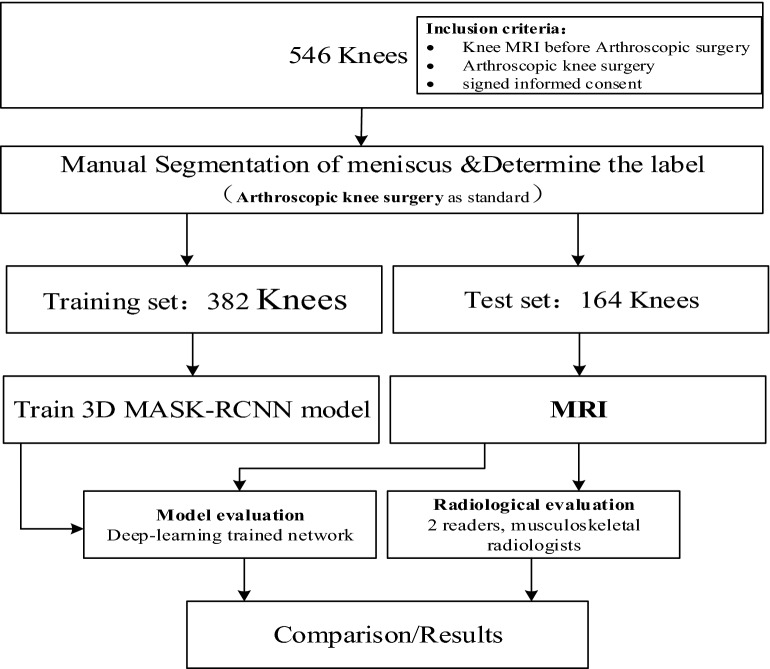
Diagram of the study design

Two radiologists and software based on Deep Convolution Neural Network (DCNN) made a retrospective evaluation of clinical patients' knee MRI to detect medial and lateral meniscus tears. All included patients underwent knee arthroscopy, and the meniscus was evaluated after MRI before knee arthroscopy. This study uses the knee joint operation report as a reference standard. The results of DCNN (Deep Convolution Neural Network) model and radiology diagnosis were compared, and the difference in diagnostic performance was calculated. The flowchart of the study design is displayed in Fig. 1.

#### Source of data set

This retrospective study was approved by the Ethics Review Committee of the Second Affiliated Hospital of Fujian Medical University, with Ethics No. 303 of [2021]. Information about patients was strictly confidential. Between July 2013 and July 2014, 546 knees with meniscal injuries were recorded from 533 patients admitted to the Department of Orthopedics of the second Affiliated Hospital of Fujian Medical University. All patients had arthroscopically confirmed meniscus injury and underwent MRI before arthroscopy of the affected knee. Among 533 patients, 336 were males, and 197 were females, ranging in age from 9 to 78 years, with an average of 51.3 ± 10.5 years. Knees with meniscus injury were divided into meniscus tear and non-tear of the meniscus. Among 546 knees with meniscus injuries, 331 knees with meniscus tears and 215 knees with non-tears of meniscus were found. Results of arthroscopy surgery were obtained by orthopedic specialists experienced in arthroscopic surgery. Patients' MRI images of their menisci were divided into a training set and a validation set (7:3). Stratified random sampling was used both in training and validation sets to maintain the same proportions of non-tears and tears.

Inclusion criteria were as follows: Meniscus that has been diagnosed arthroscopically and have been scanned with an MRI.

Exclusion criteria included the following: unavailable or incomplete clinical or MRI information; poor quality MRI images with a low signal-to-noise ratio (SNR);

Acquisition of MRI images and arthroscopic images. A dedicated 15-channel transmitting/receiving knee joint coil from Philips 3.0 T MRI system was used to examine all patients. Instructed to maintain the static alignment of the knee during scans in the coronal and sagittal planes, the patient was fixed with a sandbag on the lower leg. The sagittal scanning line was perpendicular to the tibial plateau, the coronal scanning line paralleled to the articular space, and the scanning center horizontal to the articular space. Our study focuses on Sagittal PDW sequence. The parameters of scanning are as follows: TR/TE = 3000 ms/30 MS, matrix = 256 × 256, average number of times = 1, and flip angle = 30°. Layer spacing and thickness were 1 and 3 mm, respectively. Arthroscopy: the operating system for arthroscopy was digitalized by Smith & Nephew, UK.

**Fig. 2:**
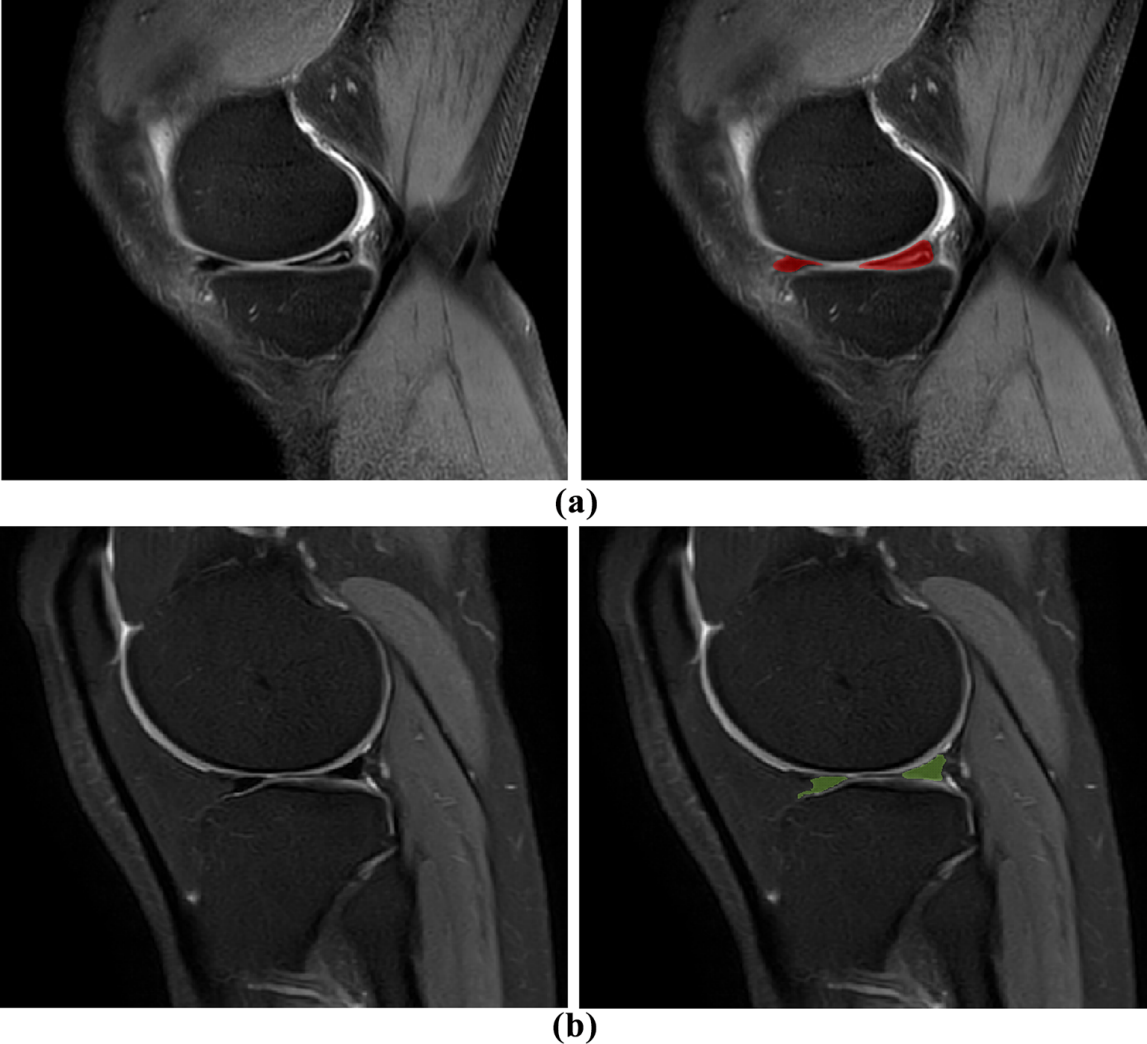
MRI PDW images of the right knee joint. **a** Tear of the posterior horn of the medial meniscus of the right knee. **b** None-tear meniscus of the right knee joint; the red and green areas are manually segmented meniscus images

A radiologist (10 year experience radiologist was blinded to the findings or the results of arthroscopic surgery.) independently completed each ROI using ITK-SNAP 3.6.0, and each sagittal section of the ipsilateral meniscus was manually segmented (Fig. 2). Labels of meniscal tears were based on arthroscopic reports from the same group of orthopedic surgeons as a standard of reference. For each knee, as long as one side of the meniscus was a tear, the knee was judged as a meniscus tear.

## Mask RCNN-based model

### 3D-Mask RCNN architecture

**Fig. 3:**
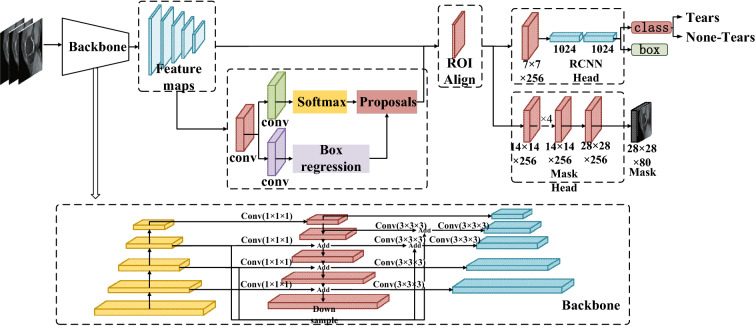
Architecture of 3D Mask RCNN

A 3D-mask RCNN-based model for the detection and segmentation of meniscus tears was developed (Fig. 3). The Mask RCNN is an extension of Faster RCNN, which detects rectangular objects as a regression and classification task [15]. The object mask is produced by another branch of the RCNN [16]. Since the 3D convolution kernels in the network require a lot of memory, we use small image regions (called patches) of size 256 × 256 × 24. To obtain an accurate model for the detection of meniscal tears, these patches are used to train a 3D-Mask RCNN. To reconstruct the entire sagittal PDW sequence for knee MRI, patches from the test set were then applied to the model, and the resulting reconstruction was then reassembled. The meniscal tear probability for MRI was calculated using the projected likelihood of each patch, and the bounding box was utilized to determine the potential meniscal area.

The original mask RCNN model was modified to a 3D version (Fig. 3). In the network, feature pyramids of different scales are extracted using the Residual Network (ResNet)-Feature Pyramid Network (ResNet-FPN) backbone [17, 18]. With FPN (Feature Pyramid Network), top-down and bottom-up features are combined at different levels. ResNet has a 50-layer deep structure in stage 4 (ResNet-50-C4). With ResNet and FPN together, the extraction of features is more accurate and faster. The candidate bounding boxes are generated from input images using a Region Proposal Network (RPN). The extracted feature maps were aligned with the inputs using a quantization-free layer, namely, ROIAlign. Layers such as this reduce misalignments between ROIs and extracted features in RoIPool. The detection branch uses a classifier network and bounding box regression to determine the box probability and location of each proposed ROI. Using a fully connected network (FCN), the mask branch derives probability and location information from the feature maps to predict segmentation masks for each ROI. For all layers, the Rectified Linear Unit (ReLU) is used as the activation function.

### 3D Mask RCNN Training

Training the 3D-Mask RCNN used He et al.'s initialization strategy [19], which was effective and was developed using Adam optimizer [20]. Only positive ROI (Region of interest) was considered when defining mask loss for meniscal segmentation. Initially, the learning rate was 0.001, which decreased by a factor of 0.5 after every 100 epochs. During training, this learning rate was changed to increase performance and training speed. An object mask branch has been added to RCNN for the prediction of object masks. There are 32 ROIs per mini-batch. The entire network will be trained in the same end-to-end manner as the RPNs.

Faster RCNN weights are initially initialized randomly with a zero-mean Gaussian distribution compared to our framework. Experimentally determined initial learning rates were set at 0.001 for all layers, and decreased by 0.5 after every 50 epochs. There are two mini-batch images per GPU with ROIs of 256 samples each. The loss function is divided into classification loss and bounding box loss, and Smooth L1 regularization is defined in Ren et al [15]. End-to-end training of jointly trained RPN and Faster RCNN is performed to train the entire network.

### Performance analysis

The detected target is compared to the real meniscus marked by an experienced radiologist. More specifically, experienced radiologists manually annotated 3D bounding boxes, and true positive (TP) objects were represented by ROIs extracted from locations marked by radiologists. Background or non-meniscal regions were marked as negative cases. For meniscus segmentation, if its intersection with the true meniscus (IOU) is greater than 50%, the test is determined to be TP. The ratio of positive and negative ROI is 3 to 2.

FROC curves are defined as plots of sensitivity against the average number of false positives per joint of the knee. Compute FROC curves by changing the threshold for confidence in object predictions [21].

#### Evaluation of meniscus tear classification performance of DCNN

The meniscus is divided into medial and lateral meniscus, and bilateral meniscus as a sample. The results of the test set are compared to arthroscopy results, a ROC curve is drawn, and AUC is calculated.

Image segmentation was evaluated using Dice coefficient. Dice is an ensemble similarity measure function commonly used to determine how similar two samples are.

$$Dice\;(A,B) = \frac{{2\left| {A \cap B} \right|}}{\left| A \right| + \left| B \right|}$$

Among them, A is the delineated meniscus area, B is the meniscus area obtained by algorithm segmentation, and the value of Dice is from 0 to 1. The closer the value is to 1, the better the segmentation effect and the more accurate the model.

MRIs of the knees of the test set were independently assessed by two musculoskeletal radiologists who were both full-time and fellowship-trained. (Reader 1: YW, Radiologist with 15 years of experience in musculoskeletal radiology; reader 2: LQQ, Radiologist with 31 years of experience in musculoskeletal radiology) without knowledge of the arthroscopic surgical diagnosis. When there was a discrepancy between them, a consultation must be held to determine the final diagnosis. A state-of-the-art picture archiving system was used to evaluate anonymized data sets once personal or clinical information had been removed under radiological reading room conditions. The readers were blinded to the patients' clinical histories, intraoperative findings, or indications for knee surgery. The classification standard of meniscus injury refers to the Stoller classification method [22]. For each knee, medial and lateral meniscus were evaluated together for the presence or absence of a meniscus tear. As long as one side of the meniscus was torn, the knee was positive for the meniscus tear. According to Stoller's grade and the results of arthroscopic knee surgery, ROC curve was drawn, and AUC was calculated for the radiological evaluation. To compare the DCNN model evaluation with the radiological evaluation without assuming parameters, the resulting output scores were resampled using a bootstrap test. Compare the distribution of the bootstrap variance measure was compared with the observed variance of the measure. Differences between methods were considered significant if the width of the calculated distribution was much smaller than the observed metric. The statistical significance of the performance difference between our DCNN model evaluation and the radiological evaluation was estimated from the ROC curve [23].

## Results

### Detection and segmentation performance

**Fig. 4:**
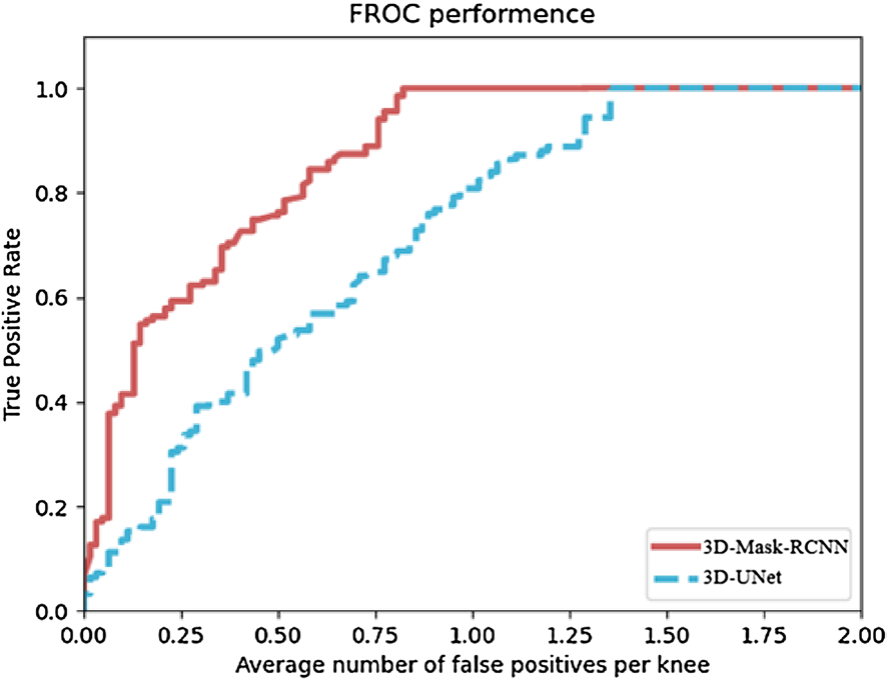
Comparison of FROC curves of 3D-Mask-RCNN and 3D-Unet

The 3D-MASK-RCNN was trained to detect and segment menisci in all of the test samples in the testing set (164 menisci). With 0.758 FPs/knee, our DCNN model was 90% sensitive and 95% sensitive with 0.823 FPs/knee. A 3D-UNET model with completely consistent data was trained and tested against the performance of our model. Table 1 and Fig. 4 show the mean numbers of FPs per knee at different sensitivities based on FROC curves. The difference in the areas between 3D-Mask-RCNN FROC and 3D-Unet FROC was statistically significant (*p* = 0.0011) using the bootstrap test. These results indicate that the performance of 3D-Mask-RCNN for segmentation is significantly superior to that of 3D-Unet (Table 2).

**Table 1:** Comparison of the average number of Fps per knee at different sensitivities between FROC of 3D-Mask-RCNN and 3D-Unet

Sensitivity (%)	Average number of FP per knee	
	3D-UNET	Our 3D-Mask RCNN
60	0.692	0.273
70	0.837	0.370
80	0.950	0.563
90	1.272	0.758
95	1.355	0.823
Dice	0.891	0.924

**Table 2:** Bootstrap test of FROC between 3D-Mask-RCNN and 3D-Unet

FROC	FOM	CI	*p*-value
3D-Mask-RCNN	0.3732	(0.0975,0.7506)	0.0011
3D-Unet			

Figures-of-merit (FOM) is the difference in the area under FROC curve between the two methods at a threshold of 4 FPs/view; *CI* confidence interval

**Fig. 5:**
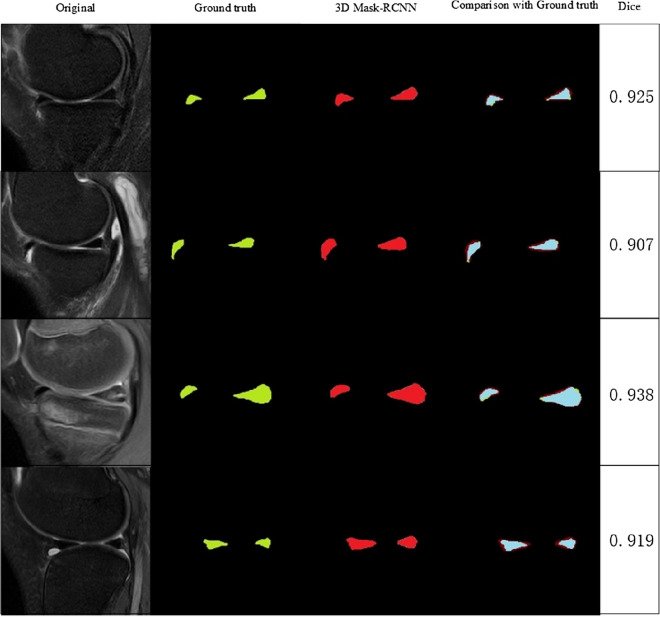
Sample of segmentation results in the meniscus region. Compared with the ground truth, the light blue is the overlapping region

The segmentation was evaluated using Dice coefficient. Our DCNN model achieved a Dice of 0.924, while the 3D-Unet model achieved a Dice of 0.891.The prediction effect can be observed from the prediction map compared to the label mask, validating the effectiveness of our method. Figure 5 depicts the sample of segmentation results in the meniscus region.

### Classification performance

The 164 samples of the test set were graded according to the Stoller [22] classification method of meniscus injury, and the meniscus injury was diagnosed by arthroscopy. To quantify the Stoller grade index for meniscus diagnosis, the knee images were divided into four grades according to the Stoller grade. According to the diagnostic experience of two radiologists, cases of level 1 will get an average probability score greater than 0 and less than or equal to 0.25, cases of level 2 will get an average probability score of greater than 0.25 and less than or equal to 0.5, and cases of level 3 will get an average probability score of With a mean probability score of greater than 0.5 and less than or equal to 0.75, cases of grade 4 will receive a mean probability score of greater than 0.75 and less than or equal to 1.00. Finally, we use the quantized mean probability score to draw the ROC curve and compare it with the ROC curve of the 3D MASK RCNN. The results of comparison are as follows (Table 3). In the Stoller grading of MRI, the accuracy rate of arthroscopic meniscus tear corresponding to the meniscus injury grade III was 0.846 and grade II was 0.66. From the analysis of the whole table, there were 11 patients with MRI grade II injury and arthroscopic tear of the meniscus, and 16 with MRI grade III injury but no meniscus tear under arthroscopy. Consequently, the overall accuracy of MRI grading of knee injury compared with arthroscopic diagnosis results was 0.835, the sensitivity was 0.889, and the specificity was 0.754.

**Table 3:** Comparison of Stoller grading of MRI and Arthroscopy results

MRI classification (Stoller [22] grade)	Arthroscopy results		Total
	Meniscus tear	Meniscus none-tear	
**0**	0	15	15
**I**	0	12	12
**II**	11	22	33
**III**	88	16	104
Total	99	65	164

**Fig. 6:**
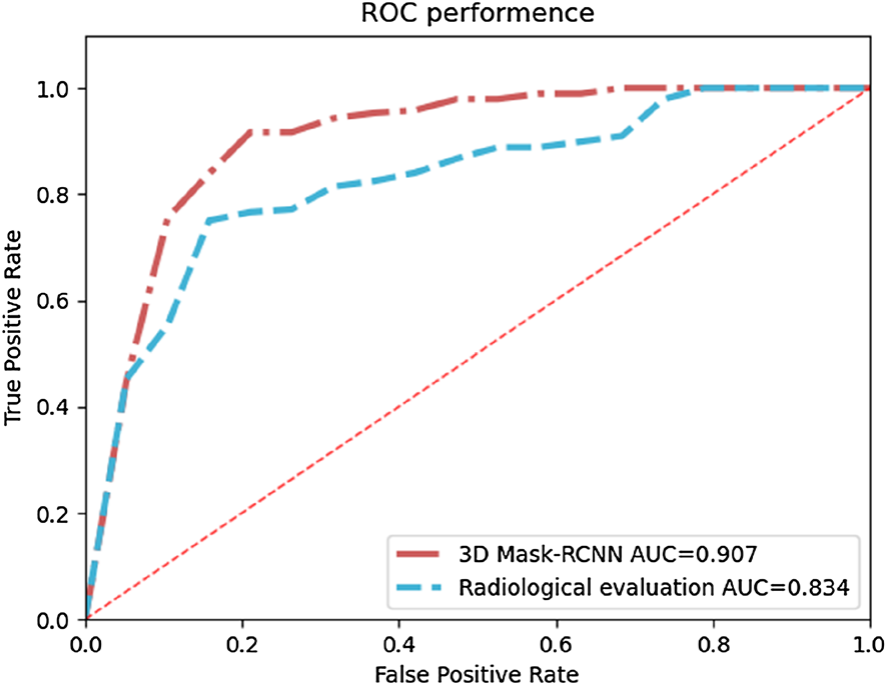
ROCs of our DCNN model and radiological evaluation

Figure 6 displays two ROCs of our 3D Mask-RCNN model and radiological evaluation. In DCNN model, AUC value, accuracy, sensitivity, and specificity values in the test set were 0.907, 0.924, 0.941, and 0.785, respectively. In radiological evaluation, AUC value, accuracy, sensitivity, and specificity values in the test set were 0.834, 0.835, 0.889, and 0.754, respectively.

The difference in the areas between 3D-Mask-RCNN ROC and the radiological evaluation ROC was statistically significant (*p* = 0.0009) by bootstrap test. The results show that 3D Mask RCNN significantly outperforms radiological evaluation (Table 4).

**Table 4:** Bootstrap test of ROC between 3D-Mask-RCNN and 3D-Unet

ROC	FOM	CI	*p*-value
3D-Mask-RCNN	0.4237	(0.1075,0.8406)	0.0009
Radiological evaluation			

FOM is the difference in the area under FROC curve between the two methods at a threshold of 4 FPs/view; *CI* confidence interval

## Discussion

Few studies use deep learning methods to segment structures in the knee area. The researches on knee MRI meniscus segmentation algorithm based on deep convolutional neural network in recent years are listed and compared with our algorithm in Table 5. In the first of these studies, conducted by Aldrin in 2017, 2D U-net fully convolutional networks (2D UNET) and the random forest methods were comparatively applied for meniscus segmentation [24]. Their 2D U-net network reached a Dice of 0.752 and a sensitivity of 0.796. Norman et al. presented a 2D U-net for automated cartilage and meniscus segmentation of knee MR imaging data to determine relaxometry and morphometry [25]. These models produced strong Dice coefficients, particularly for 3D-DESS images, ranging between 0.770 and 0.878 in the cartilage compartments to 0.809 (lateral meniscus) and 0.753 (medial meniscus). In another study, conducted in 2018 and using convolutional nets, segmentation was performed on knee MR images of medial and lateral menisci. In this study, convolution networks and statistical surface models are used together. Based on measurements using Dice similarity criterion, 83.8% for the inner meniscus and 88.9% for the outer meniscus were achieved. Because the study was performed only in the sagittal plane DESS format MR images, its success in different excitation sequences is unknown [26]. This study proposed a 3D Mask RCNN meniscus segmentation and meniscus tear detection framework. 3D-Mask RCNN is superior to 3D-Unet for meniscus segmentation performance. Our method has achieved a Dice of 92.4% for bilateral meniscus and a sensitivity of 95% with 0.823 FPs/knee for detection performance, which significantly outperformed the 3D-Unet. Moreover, our algorithm is aimed at the conventional sagittal PDW sequence of knee MRI, not at special sequences, which may be more suitable for a wide range of clinical applications.

**Table 5:** Meniscus segmentation studies in deep Learning

Author(s) and years	Data size	Method(s)	Performance
Aldrin et al*.* [24] & 2017	18	2D U-net	Dice = 0.752 Sensitivity = 0.796
Norman et al. [25] & 2018	638	2D U-net	Dice for medial meniscus = 80.9% Dice for lateral meniscus = 75.3%
Tack A et al. [26] & 2018	552	3D U-net	Dice for medial meniscus = 83.8% Dice for lateral meniscus = 88.9%
Our 3D Mask-RCNN	546	3D Mask-RCNN	Dice for bilateral meniscus = 92.4%

Our method's performance in detecting meniscus tears has also presented great results with AUC value, accuracy, sensitivity, and specificity at 0.907, 0.924, 0.941, and 0.785, respectively, and the results of the bootstrap test shows that 3D Mask RCNN significantly outperforms radiological evaluation (Table 4). As indicated in Table 3, due to the influence of the scanning environment and the poor cooperation of patients, MRI imaging may produce various artifacts and adverse factors, so that the radiologist cannot make a correct image diagnosis. In addition, some degenerative lesions, such the fibrillation of the meniscus, which can cause the fibrillation or brush-like changes of the free edge of the meniscus, are difficult to distinguish from meniscal tears on MRI, and some tiny tears at the edges of the meniscus are difficult to identify on images and can only be identified under arthroscopy [27–29]. As a result, in the Stoller grading of MRI, the accuracy rate of arthroscopic meniscus tear corresponding to the meniscus injury grade III was 0.846 and grade II was 0.66, AUC value, accuracy, sensitivity, and specificity of radiological evaluation were only 0.834, 0.835, 0.889, and 0.754, respectively. Consequently, the most important finding of this study is that our algorithm is required to assist radiologists in improving the accuracy and efficiency of diagnosis, and it can provide effective diagnostic indicators for orthopedic surgeons before arthroscopic surgery, thereby contributing to further promotion of precise treatment.

## Conclusions

We proposed that a 3D Mask RCNN meniscus segmentation and tear detection of knee MRI. A comparison of 3D-Mask RCNN and 3D-Unet methods. We proved that 3D-Mask RCNN has a better segmentation performance. A comparison of tear detection performance with radiological evaluation that has presented the accuracy and sensitivity of 3D-Mask RCNN were significantly outperformed. In conclusion, our 3D-Mask RCNN can accurately segment meniscus and perform tear detection in a fully automated manner with higher accuracy than musculoskeletal radiologists can reduce unnecessary arthroscopic examination of patients, and promote accurate treatment of meniscus tear by reducing excessive surgical treatment.

## Data Availability

Data are available on request from the authors due to privacy/ethical restrictions.

## References

[1] Kawahara T, Sasho T, Katsuragi J, Ohnishi T, Haneishi H (2017). Relationship between knee osteoarthritis and meniscal shape in observation of Japanese patients by using magnetic resonance imaging. J Orthop Surg Res.

[2] Englund M, Guermazi A, Gale D, Hunter DJ, Felson DT (2008). Incidental meniscal findings on knee MRI in middle-aged and elderly persons. N Engl J Med.

[3] Karpinski K, Petersen W (2017). Beidseitiger Horizontalriss des Innen- und Außenmeniskus nach Hyperextensionstrauma. Arthroskopie.

[4] Santiago (2018). Meniscal root tears: current concepts review. Arch Bone Jt Surg.

[5] Lecouvet F (2018). Magnetic resonance imaging (MRI) of the knee: identification of difficult-to-diagnose meniscal lesions. Diagn Interv Imaging.

[6] Naraghi AM, White LM (2016). Imaging of athletic injuries of knee ligaments and menisci: sports imaging series. Radiology.

[7] Ruth C, Gayle W, Stephen B, Nicola M (2006). Magnetic resonance imaging versus arthroscopy in the diagnosis of knee pathology, concentrating on meniscal lesions and ACL tears: a systematic review. Br Med Bull.

[8] Porter M, Shadbolt B (2021). Accuracy of standard magnetic resonance imaging sequences for meniscal and chondral lesions versus knee arthroscopy. A prospective case-controlled study of 719 cases. ANZ J Surg.

[9] Zhang B, Zhang Y, Cheng H, Xian M, Gai S, Cheng O, Huang K (2018) Computer-aided knee joint magnetic resonance image segmentation - a survey. ArXiv, abs/1802.04894

[10] Mazurowski MA, Mateusz B, Ashirbani S, Bashir MR (2019). Deep learning in radiology: an overview of the concepts and a survey of the state of the art with focus on MRI. J Magn Reson Imaging.

[11] Filippo (2018). Artificial intelligence in medical imaging: threat or opportunity? Radiologists again at the forefront of innovation in medicine. Eur Radiol Exp.

[12] Chen H, Zhang X, Wang X, Quan X, Zhao Y (2021). MRI-based radiomics signature for pretreatment prediction of pathological response to neoadjuvant chemotherapy in osteosarcoma: a multicenter study. Eur Radiol.

[13] Ubaldi L, Valenti V, Borgese RF, Collura G, Marrale M (2021). Strategies to develop radiomics and machine learning models for lung cancer stage and histology prediction using small data samples. Physica Med.

[14] Yy A (2021). Magnetic resonance imaging radiomics signatures for predicting endocrine resistance in hormone receptor-positive non-metastatic breast cancer. The Breast.

[15] Ren S, He K, Girshick R, Sun J (2017). Faster R-CNN: towards real-time object detection with region proposal networks. IEEE Trans Pattern Anal Mach Intell.

[16] He, K., Gkioxari, G., Dollár, P. & Girshick, R. Mask R-CNN. IEEE Transactions on Pattern Analysis & Machine Intelligence. 2017. 10.1109/TPAMI.2018.2844175 29994331

[17] Lin, T. Y. et al. Feature Pyramid Networks for Object Detection. 2017 IEEE Conference on Computer Vision and Pattern Recognition (CVPR). 2017.

[18] He, K., Zhang, X., Ren, S. & Sun, J. in 2016 IEEE Conference on Computer Vision and Pattern Recognition (CVPR). 2016.

[19] He, K., Zhang, X., Ren, S. & Sun, J. Delving Deep into Rectifiers: Surpassing Human-Level Performance on ImageNet Classification. CVPR. 2015.

[20] Kingma, D. P. & Ba, J. Adam: A Method for Stochastic Optimization. arXiv e-prints. 2014.

[21] Bandos AI, Rockette HE, Song T, Gur D (2009). Area under the free-response ROC curve (FROC) and a related summary index. Biometrics.

[22] Stoller DW, Martin C, Crues J, Kaplan L, Mink JH (1987). Meniscal tears: pathological correlation with MR imaging. Radiology.

[23] Bornefalk H, Hermansson AB (2005). On the comparison of FROC curves in mammography CAD systems. Med Phys.

[24] Aldrin, F. Automated Segmentation of the Meniscus. 2017.

[25] Norman (2018). Use of 2D U-net convolutional neural networks for automated cartilage and meniscus segmentation of knee MR imaging data to determine relaxometry and morphometry. Radiology.

[26] Tack A, Mukhopadhyay A, Zachow S (2018). Knee menisci segmentation using convolutional neural networks: data from the osteoarthritis initiative. Osteoarthritis Cartilage.

[27] Guo, J. M., Liu, P. C. & Zhang, W. T. MRI Diagnosis of Meniscal Injuries of the Knee:Correlated with Arthroscopy. Radiologic Practice (2009).

[28] Christian (2016). Diagnostic efficacy of 3-T MRI for knee injuries using arthroscopy as a reference standard: a meta-analysis. AJR Am J Roentgenol.

[29] Wang CW, Liu LB, Jia WD, Zhao B, Zheng H (2014). A comparative analysis of MRI and arthroscopy in meniscus injury of the knee joint. Chin J Tissue Eng Res.

